# Clinical Significance of the IL-33/sST2 Axis and Vitamin D Status in the Assessment of Disease Severity and Exacerbation Risk in Asthma: A Prospective Controlled Study

**DOI:** 10.3390/jcm15103663

**Published:** 2026-05-10

**Authors:** Mine Huryasar Eskici, Nilgun Basaran, Mukaddes Goker, Buse Akyol, Gulcan Guntas

**Affiliations:** 1 Department of Clinical Biochemistry, Prof. Dr. Cemil Tascioglu City Hospital, 34384 Istanbul, Turkey; mine.huryasar@gmail.com (M.H.E.); basarannilgun@gmail.com (N.B.); buseakillilar@gmail.com (B.A.); 2 Department of Pulmonology, Prof. Dr. Cemil Tascioglu City Hospital, 34384 Istanbul, Turkey; mukadder@yahoo.com; 3 Department of Medical Biochemistry, School of Medicine, Atlas University, 34403 Istanbul, Turkey

**Keywords:** asthma, IL-33, soluble ST2, vitamin D, type 2 inflammation, biomarkers

## Abstract

**Background:** Asthma is a heterogeneous chronic inflammatory airway disease characterized by recurrent exacerbations and variable airflow limitation. Epithelial-derived alarmins, particularly interleukin-33 (IL-33) and its receptor ST2, play key roles in type 2 inflammation. The soluble form of ST2 (sST2) acts as a decoy receptor regulating IL-33 signaling. Vitamin D is an important immunomodulator influencing airway inflammation, but its interaction with the IL-33/ST2 pathway remains unclear. **Objective:** To evaluate the association between serum IL-33, sST2, and 25-hydroxyvitamin D [25(OH)D] levels with asthma severity and exacerbation status, and to assess their potential as clinical biomarkers. **Methods:** This study enrolled 52 adult asthma patients (27 experiencing exacerbation and 25 in remission) and 28 healthy controls. Serum levels of IL-33 and sST2 were measured using enzyme-linked immunosorbent assays, while 25(OH)D concentrations were determined via electrochemiluminescence immunoassay. **Results:** Serum sST2 levels were significantly higher and 25(OH)D levels significantly lower in asthma patients compared with controls (*p* < 0.000 for both). Serum IL-33 levels did not differ significantly between groups (*p* > 0.05). During exacerbation, sST2 levels were markedly elevated compared with remission (*p* < 0.001), whereas vitamin D levels were significantly reduced (*p* = 0.038). A significant negative correlation was identified between sST2 and 25(OH)D (r = −0.333, *p* = 0.016). **Conclusions:** The presence of asthma and the severity of exacerbations are associated with elevated circulating sST2 levels and reduced vitamin D levels. These findings suggest a regulatory interaction between vitamin D and the IL-33/ST2 axis in airway inflammation and indicate that targeting this axis could be a potential therapeutic strategy.

## 1. Introduction

Asthma is a heterogeneous chronic inflammatory airway disease characterized by variable airflow limitation, bronchial hyperresponsiveness, and recurrent exacerbations affecting approximately 300 million individuals worldwide [1]. Despite advances in targeted biologic therapies, asthma remains a major contributor to global morbidity and healthcare utilization. Contemporary concepts define asthma as a syndrome composed of distinct inflammatory endotypes rather than a single disease entity [2].

Type 2 inflammation is the dominant immunopathological mechanism in many asthma patients. This process involves T helper type 2 (Th2) lymphocytes, group 2 innate lymphoid cells (ILC2), eosinophils, mast cells, and basophils, leading to the secretion of interleukin (IL)-4, IL-5, and IL-13 [3,4]. These cytokines promote immunoglobulin E (IgE) production, mucus hypersecretion, airway remodeling, and bronchial hyperreactivity. Increasing attention has focused on epithelial-derived alarmins as upstream regulators initiating allergic airway inflammation [5,6,7].

It is suggested that the airway epithelium is a key organ for immune function, which releases epithelial-derived cytokines (thymic stromal lymphopoietin (TSLP), IL-33 and IL-25) that likely play an important role in asthma pathogenesis [6,7].

IL-33, a member of the IL-1 cytokine family, functions as an alarmin released following epithelial injury caused by allergens, infections, or environmental stress [5,8]. IL-33 signals through the transmembrane receptor ST2L and activates nuclear factor-κB and mitogen-activated protein kinase pathways, resulting in amplification of type 2 immune responses [8,9]. Experimental asthma models demonstrate that inhibition of IL-33 signaling reduces eosinophilic inflammation and airway hyperresponsiveness [10].

The soluble isoform of ST2 (sST2) acts as a circulating decoy receptor, interfering with the IL-33/ST2 axis and sequestering IL-33 [10,11]. Increased circulating sST2 levels have been associated with inflammatory diseases and tissue injury and may reflect compensatory anti-inflammatory mechanisms [11,12]. Clinical studies evaluating asthma patients have suggested an association between elevated sST2 levels and exacerbation risk, although findings remain inconsistent [11,13].

Vitamin D has emerged as an important regulator of immune homeostasis beyond its classical role in calcium metabolism. Active vitamin D modulates dendritic cell maturation, suppresses Th1 differentiation, enhances regulatory T-cell responses, and promotes IL-10 production via vitamin D receptor signaling [14,15,16]. Epidemiological and meta-analytic studies have demonstrated that vitamin D deficiency is associated with poor asthma control, increased exacerbation frequency, and corticosteroid resistance [17,18,19].

Experimental studies further suggest a mechanistic interaction between vitamin D signaling and the IL-33/ST2 pathway. Vitamin D enhances the expression of sST2 in airway epithelial cells and lymphocytes, thereby inhibiting the IL-33/ST2L axis and its type 2 inflammatory effects [20,21]. However, clinical studies evaluating the combined relationship between vitamin D status and IL-33/sST2 signaling in asthma severity remain limited.

To the best of our knowledge, the present study is among the limited prospective controlled clinical investigations in adults to simultaneously evaluate circulating IL-33, sST2, and 25-hydroxyvitamin D concentrations across three distinct disease states: exacerbation, remission, and healthy controls. Although prior experimental work has demonstrated that vitamin D upregulates sST2 production in vitro, and pediatric studies have explored correlations between these markers, a prospective integrated clinical assessment of the IL-33/sST2 decoy receptor axis alongside vitamin D status in adult asthma patients has been limited. The present study therefore addresses this gap by simultaneously assessing the clinical relevance and biomarker utility of these three interrelated mediators in asthma disease activity and exacerbation risk.

The aim of our study was to evaluate serum interleukin-33 (IL-33), soluble ST2 (sST2), and 25-hydroxyvitamin D (25(OH)D) levels in asthma patients during exacerbation and remission periods and to investigate their associations with disease severity and exacerbation risk, as well as their potential as clinical biomarkers.

## 2. Materials and Methods

### 2.1. Study Design and Ethical Approval

This prospective observational controlled study was conducted at the Department of Chest Diseases, Prof. Dr. Cemil Taşcıoğlu City Hospital, between 1 September and 31 December 2020. The study protocol complied with the principles of the Declaration of Helsinki and received approval from the Institutional Ethics Committee (Decision No: 314; 14 July 2020). Written informed consent was obtained from all participants prior to enrollment.

A total of 102 individuals were initially screened for eligibility. Of these, 17 were excluded to ensure age- and sex-matched group composition, and 5 were excluded due to sample hemolysis rendering laboratory analysis unreliable. The final study population comprised 80 participants: 27 asthma patients during exacerbation, 25 asthma patients in remission, and 28 healthy controls.

### 2.2. Study Population

Participants aged between 20 and 65 years were consecutively recruited from outpatient and emergency clinic admissions. The study population consisted of asthma patients and healthy volunteers, who were divided into two main groups.

Asthma diagnoses were made according to Global Initiative for Asthma (GINA) criteria (1), based on patients’ medical histories, spirometric findings, and clinical evaluations by a pulmonologist (1). The asthma group included 52 patients: 27 patients evaluated during acute exacerbation and 25 patients evaluated during remission. A total of 28 age- and sex-matched healthy volunteers constituted the control group. Asthma exacerbation was defined as worsening respiratory symptoms requiring intensified treatment or an unexpected clinical evaluation according to guideline recommendations (1).

### 2.3. Inclusion Criteria

Participants were eligible if they met the following criteria:

- Age between 20 and 65 years;
- Provision of written informed consent;
- Physician-diagnosed asthma based on GINA criteria (for the patient group);
- Normal hemogram and total IgE laboratory findings within the previous month (for healthy controls).

### 2.4. Exclusion Criteria

Participants were excluded if any of the following conditions were present:

- Active smoking;
- Vitamin D supplementation within the previous three months;
- Oral corticosteroid or monoclonal antibody therapy;
- Active infection or recent infectious disease;
- Pregnancy;
- Age below 20 or above 65 years;
- Concomitant respiratory diseases including COPD or emphysema;
- Autoimmune disorders;
- Thyroid diseases;
- Malignancy or chronic systemic diseases potentially affecting inflammatory biomarkers.

Patients using inhaled corticosteroids were not excluded, as most asthma patients, particularly those presenting during exacerbation, were already receiving regular inhaled corticosteroid therapy as part of their standard controller treatment at the time of clinical evaluation.

### 2.5. Sample Collection and Processing

Blood samples were collected from all participants, including asthma patients and healthy controls, within the same September–December period, ensuring that any seasonal influence on 25(OH)D concentrations was uniformly distributed across all three study groups. Following an overnight fasting period of at least eight hours, venous blood samples were collected between 08:00 and 10:00 AM using serum separator gel tubes (BD Vacutainer, Plymouth, UK). Samples were allowed to clot at room temperature for 15 min and subsequently centrifuged at 2860× *g* for 10 min. Serum aliquots were divided into three equal Eppendorf tubes and stored at −80 °C until analysis. Hemolyzed, lipemic, or icteric samples were excluded to ensure analytical reliability.

### 2.6. Laboratory Measurements

Serum IL-33 concentrations were measured using a commercially available enzyme-linked immunosorbent assay (Human IL-33 ELISA kit; Invitrogen, Vienna, Austria). The assay measurement range was 7.8–500 pg/mL with a sensitivity of 0.2 pg/mL and intra-assay coefficient of variation of 4.7%.

Serum-soluble ST2 concentrations were determined using a Human ST2 (IL-33 receptor) ELISA kit (Invitrogen, Vienna, Austria). The assay measurement range was 4.7–300 pg/mL with a sensitivity of 4.6 pg/mL and an intra-assay coefficient of variation of 4.5%.

The Reader Biotek ELx800 (Bio-Tek Instruments, Winooski, VT, USA) was used for readings, and the KcJunior software (Bio-Tek Instruments, Winooski, VT, USA) was used.

Serum 25-hydroxyvitamin D [25(OH)D] concentrations were measured using an electrochemiluminescence immunoassay method on the Roche Cobas e801 analyzer (Roche Diagnostics, Mannheim, Germany) employing the Elecsys Vitamin D Total II kit. The intra-assay and inter-assay coefficients of variation were <7.6% and <8.9%, respectively.

Total IgE concentrations were determined using a sandwich electrochemiluminescence immunoassay (Elecsys IgE II kit, Roche Diagnostics, Mannheim, Germany). The analytical precision demonstrated intra-assay and inter-assay coefficients of variation below 4%.

### 2.7. Pulmonary Function Tests

Spirometry was performed using standardized pulmonary function testing procedures in accordance with international respiratory society recommendations. Forced expiratory volume in one second (FEV1), forced vital capacity (FVC), and FEV1/FVC ratio were recorded.

### 2.8. Statistical Analysis

Sample size was determined a priori using GPower 3.1.9 software. Based on expected mean values derived from the literature for each primary outcome, one-way ANOVA was used as the reference test with α = 0.05 and β = 0.20 (power = 80%). For serum vitamin D, assuming group means of approximately 12, 16, and 25 ± 2 ng/mL, the estimated effect size was 0.56, requiring a minimum of 19 participants per group (total *n* = 57). For serum sST2, assuming group means of approximately 35, 80, and 100 ± 10 µg/mL, the estimated effect size was 0.54, again requiring a minimum of 19 participants per group (total *n* = 57). For serum IL-33, assuming group means of approximately 100, 200, and 400 ± 100 pg/mL, the estimated effect size was 0.53, requiring a minimum of 20 participants per group (total *n* = 60). Accordingly, the target sample was set at 80 participants (20 controls and 60 asthma patients divided equally into exacerbation and remission subgroups), consistent with the practical capacity of the 96-well ELISA plate format used. The final enrolled sample of 80 participants (28 controls, 27 exacerbation, 25 remission) met or exceeded the minimum required sample size for all three primary outcomes.

Statistical analyses were performed using IBM SPSS Statistics version 22.0 (IBM Corp., Armonk, NY, USA). Normality of distribution was assessed using the Shapiro–Wilk test. Descriptive statistics were reported as mean ± standard deviation or frequency, as appropriate. For comparisons of normally distributed parameters across three groups, one-way ANOVA was used, with Tukey’s HSD post hoc test applied to identify the source of significant differences. For non-normally distributed parameters, the Kruskal–Wallis test was used for between-group comparisons, followed by Dunn’s test for pairwise comparisons. For two-group comparisons, Student’s *t*-test was applied for normally distributed parameters and the Mann–Whitney U test for non-normally distributed parameters. Categorical variables were compared using the chi-square test with Yates’ continuity correction where appropriate. Pearson correlation analysis was used to examine relationships between normally distributed continuous variables, including sST2, IL-33, 25(OH)D, FEV1/FVC, IgE, WBC, and eosinophil counts; results are reported as Pearson r coefficients with 95% confidence intervals. Spearman’s rho correlation was applied where normality assumptions were violated. Multivariate logistic regression analysis was performed to identify independent predictors of asthma and asthma exacerbation. Receiver operating characteristic (ROC) curve analysis was conducted to evaluate the diagnostic utility of sST2 for discriminating asthma patients from healthy controls and for discriminating exacerbation from remission; the optimal cut-off value was determined based on the Youden index. Statistical significance was set at *p* < 0.05.

## 3. Results

A total of 80 participants were included in the study, consisting of 52 patients with asthma and 28 healthy controls. Among asthma patients, 27 were evaluated during exacerbation and 25 during remission.

The general characteristics of the studied groups are shown in Table 1. No statistically significant differences were observed among exacerbation, remission, and control groups in terms of age, body mass index (BMI), or sex distribution (*p* > 0.05).

**Figure 1:**
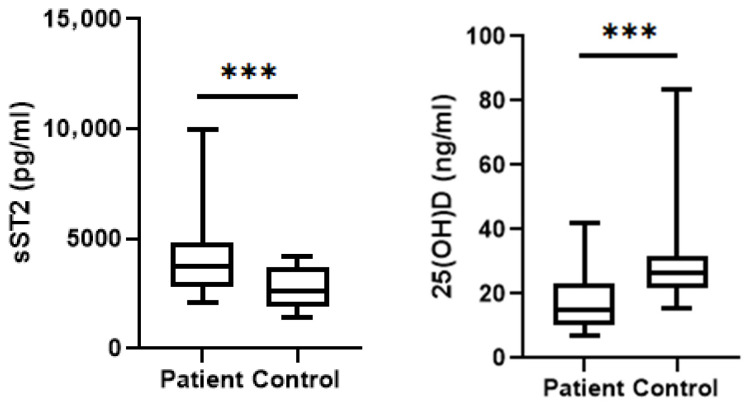
Comparison of sST2 concentrations and vitamin D levels between asthma patients and controls. Asthma patients demonstrated significantly elevated circulating sST2 concentrations and significantly lower 25(OH)D levels compared with controls (*** *p* = 0.0002 and *** *p* < 0.0001, respectively).

The evaluation of study parameters for the patient and control groups is shown in Table 2. Serum sST2 concentrations were significantly higher in asthma patients compared with the control group, whereas vitamin D levels were markedly reduced (*p* < 0.001 for both) (Figure 1). Total IgE and WBC counts were significantly elevated among asthma patients (*p* = 0.001 and *p* = 0.01, respectively).

Serum IL-33 concentrations did not differ significantly between groups (*p* > 0.005). IL-33 concentrations demonstrated a markedly non-normal distribution confirmed by the Shapiro–Wilk test (W = 0.323, *p* < 0.001), with a coefficient of variation of 165.5%, skewness of 4.808, and kurtosis of 22.71, reflecting a substantially right-skewed distribution with a median of 2.991 pg/mL but a mean of 4.614 pg/mL driven by three patients with markedly elevated values over 35 pg/mL. The majority of participants clustered near the lower end of the measurable range, with 75% of values below 3.929 pg/mL. Accordingly, non-parametric tests were applied for all between-group comparisons involving IL-33.

Significant intergroup differences were observed for sST2, vitamin D, IgE, and WBC parameters (Table 3). In the exacerbation group, sST2 levels were significantly higher and 25(OH)D levels were significantly lower than in the remission group (*p* < 0.001 for both). IgE levels and WBC counts of study groups were significantly highest in the exacerbation group (*p* = 0.001 and *p* = 0.002, respectively). There were no statistically significant differences in IL-33 levels and eosinophil amounts between three groups (*p* > 0.05).

**Figure 2:**
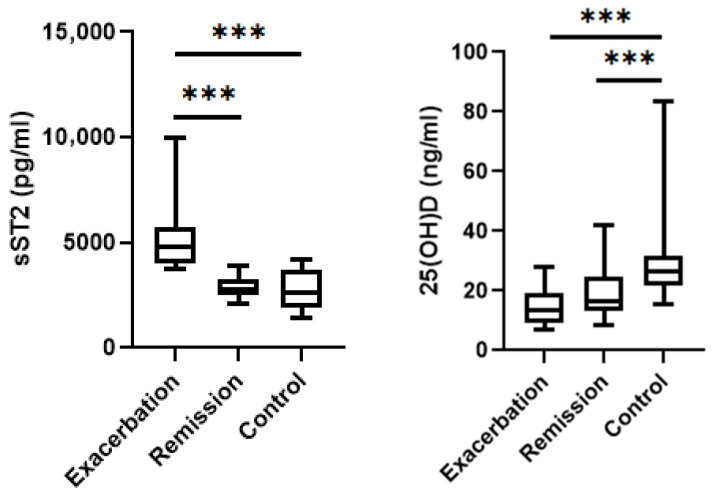
Comparison of sST2 concentrations and vitamin D levels between exacerbation, remission and controls. The exacerbation group demonstrated significantly higher circulating sST2 concentrations and significantly lower 25(OH)D levels compared with the remission and control groups (*** *p* < 0.001; horizontal lines indicate statistically significant pairwise comparisons).

Exacerbation patients demonstrated markedly elevated sST2 concentrations compared to remission and control groups (*p* < 0.001) (Figure 2). The 25(OH)D levels in the control group were found to be significantly higher than those in the exacerbation and remission groups (*p* < 0.001 and *p* = 0.003, respectively) (Figure 2).

As presented in Table 4, several parameters differed significantly between the exacerbation and remission groups. Serum sST2 concentrations were markedly elevated in patients during exacerbation compared with those in remission (*p* < 0.001), suggesting increased activation of the IL-33/ST2 signaling axis during acute disease episodes. Vitamin D levels were significantly lower in the exacerbation group than in the remission group (*p* = 0.038), consistent with a potential immunomodulatory role of vitamin D in disease activity. White blood cell counts were also significantly higher during exacerbation (*p* = 0.012), reflecting the systemic inflammatory response associated with acute asthma episodes. Furthermore, pulmonary function was significantly impaired during exacerbation, as evidenced by a markedly lower FEV1/FVC ratio compared with the remission period (*p* < 0.001), indicating the functional impact of acute airway inflammation on ventilatory capacity. In contrast, serum IL-33 concentrations did not differ significantly between the two groups (*p* > 0.05), which may reflect the limitations of circulating IL-33 as a systemic biomarker of disease activity.

Pearson correlation analyses were performed to evaluate the associations between the primary biomarkers and clinical parameters across the entire study population (Table 5). The strongest correlation observed was between sST2 and FEV1/FVC ratio (r = −0.640, *p* < 0.001), indicating that higher circulating sST2 concentrations were significantly associated with worse pulmonary function. A significant negative correlation was also identified between sST2 and 25(OH)D levels (r = −0.333, *p* = 0.016), suggesting an inverse relationship between vitamin D status and circulating sST2 concentrations. Furthermore, sST2 showed a significant positive correlation with WBC count (r = 0.285, *p* = 0.041), consistent with the association between systemic inflammatory activity and sST2 elevation. Among other parameters, IgE demonstrated significant positive correlations with both eosinophil percentage (r = 0.283, *p* = 0.042) and absolute eosinophil count (r = 0.472, *p* < 0.001), reflecting the relationship between allergic sensitization and eosinophilic inflammation. Serum IL-33 did not show a statistically significant correlation with any of the clinical parameters evaluated, including sST2 (r = 0.272, *p* = 0.051) and 25(OH)D (r = 0.190, *p* = 0.178).

**Figure 3:**
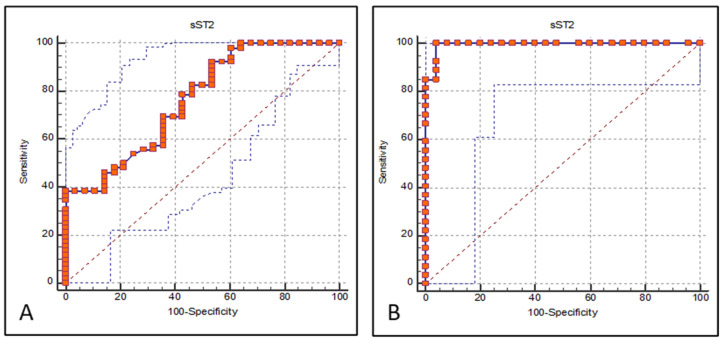
Receiver operating characteristic (ROC) curve for serum sST2 in discriminating asthma patients from healthy controls (**A**) and in discriminating patients during exacerbation from those in remission (**B**). Graph A has AUC = 0.762 (standard error = 0.06, *p* = 0.001). The optimal cut-off value was >2446.1 pg/mL, with a sensitivity of 92.3% and a specificity of 46.4%. AUC, area under the curve; sST2, soluble ST2. Graph B has AUC = 0.994 (standard error = 0.01, *p* = 0.001). The optimal cut-off value was >3733 pg/mL, with a sensitivity of 100% and a specificity of 96.0%. AUC, area under the curve; sST2, soluble ST2. Red squares represent the ROC curve data points. Red dashed lines indicate the optimal cut-off point determined by the Youden index. The blue dashed line represents the reference line of no discrimination (AUC = 0.5).

To assess the clinical biomarker utility of sST2, ROC curve analyses were performed for two clinically relevant discriminations: asthma versus healthy controls and exacerbation versus remission (Figure 3). For the discrimination of asthma patients from healthy controls, the area under the ROC curve (AUC) for sST2 was 0.762 (standard error = 0.06, *p* = 0.001), indicating acceptable discriminatory performance. The optimal cut-off value was determined as >2446.1 pg/mL, yielding a sensitivity of 92.3% and a specificity of 46.4%. For the discrimination of exacerbation from remission, sST2 demonstrated outstanding discriminatory performance, with an AUC of 0.994 (standard error = 0.01, *p* = 0.001). The optimal cut-off value was >3733 pg/mL, with a sensitivity of 100% and a specificity of 96.0%, indicating that sST2 is a highly accurate biomarker for identifying asthma exacerbation.

Multivariate logistic regression analyses were performed to identify independent predictors of asthma and asthma exacerbation. For asthma diagnosis, a model including sST2, 25(OH)D, IgE, and WBC demonstrated good overall explanatory power (Nagelkerke R^2^ = 0.546, model accuracy = 82.5%, *p* < 0.05). Within this model, sST2 (OR = 1.001, 95% CI: 1.000–1.001, *p* = 0.025), IgE (OR = 1.003, 95% CI: 1.000–1.006, *p* = 0.049), and 25(OH)D (OR = 0.870, 95% CI: 0.795–0.953, *p* = 0.003) were identified as statistically significant independent predictors, with higher sST2 and IgE levels associated with increased asthma risk and higher 25(OH)D levels associated with reduced risk (Table 6). For exacerbation status, a model including sST2, 25(OH)D, WBC, and FEV1/FVC demonstrated excellent explanatory power (Nagelkerke R^2^ = 0.929, model accuracy = 96.2%, *p* < 0.05). Only sST2 remained a statistically significant independent predictor of exacerbation (OR = 1.012, 95% CI: 0.997–1.029, *p* = 0.046), indicating that its association with acute disease activity persists independently of other inflammatory and functional parameters (Table 6).

## 4. Discussion

Asthma is increasingly recognized as a disease initiated and maintained by epithelial barrier dysfunction and alarmin-driven immune activation, rather than being solely dependent on adaptive Th2 polarization [1,2]. In this emerging model, epithelial cytokines such as IL-33 act as upstream regulators that connect environmental exposure, innate immune activation, adaptive allergic inflammation and disease exacerbations [5,8,22]. In the present study, we investigated the IL-33/ST2 signaling axis alongside vitamin D status in asthma patients, evaluating their association with disease activity. Our main finding was that circulating soluble ST2 (sST2) levels were significantly higher in asthma patients than in healthy controls (Figure 1) and increased markedly during exacerbation periods (Table 2 and Table 3). Vitamin D concentrations, on the other hand, were significantly lower, particularly during disease exacerbation (Figure 2). In contrast, serum IL-33 levels did not significantly differ between study groups (Table 3).

IL-33 acts as an upstream alarmin that activates ILC2s, mast cells, and Th2 lymphocytes via ST2L signaling, amplifying type 2 inflammatory responses and contributing to airway remodeling [8,22,23]. In this context, the significantly elevated sST2 levels observed in asthma patients in our study may represent a compensatory anti-inflammatory response aimed at limiting excessive IL-33 signaling. As a soluble decoy receptor, sST2 binds circulating IL-33 and prevents interaction with membrane-bound ST2L receptors; thereby, sST2 acts as a negative regulator of Th2 cytokine production by the IL-33 signaling [10,22]. Blocking IL-33/ST2 signaling has been shown to reduce eosinophilic infiltration, mucus production, and airway inflammation [9]. Therefore, the increased systemic sST2 concentrations detected in our patients are likely to reflect the activation of endogenous regulatory mechanisms attempting to counterbalance inflammation driven by epithelial alarmins.

Studies have shown that ST2 signaling plays a role in sustained inflammatory responses and delayed resolution following airway injury [3,10,24]. Consistent with our findings, previous studies have reported elevated levels of sST2 in the blood during inflammatory pulmonary diseases and asthma exacerbations [12,13]. The marked increase in sST2 levels observed during exacerbations may therefore suggest increased activation of the IL-33 pathway, accompanied by the production of compensatory decoy receptors. To further evaluate the independent effects of biomarkers and reduce the potential impact of confounding factors, multivariate logistic regression analyses were performed (Table 6). In the assessment of asthma risk, sST2, 25(OH)D, IgE, and WBC were included in the model, which showed good explanatory power (82.5%). Increased sST2 and IgE levels were independently associated with a higher risk of asthma, whereas higher 25(OH)D levels were associated with a reduced risk. Similarly, for exacerbation status, sST2, vitamin D, WBC, and FEV1/FVC were included in the logistic regression model (Table 6). The model demonstrated excellent explanatory power (96.2%), and only sST2 remained a statistically significant independent predictor of exacerbation. These findings suggest that the association between elevated sST2 levels and asthma exacerbation persists independently of other inflammatory and clinical parameters, further supporting the potential value of sST2 as a biomarker of disease activity and severity. The growing development of biologic therapies that target epithelial alarmins, including monoclonal antibodies such as itepekimab that target IL-33 and agents such as astegolimab that target ST2, further highlights the importance of identifying biomarkers that reflect pathway activation [25,26]. Our findings suggest that circulating sST2 may be a more stable systemic biomarker than IL-33 itself.

Despite the well-established role of IL-33 in the pathogenesis of allergic airway inflammation, our study found that, unlike sST2, serum IL-33 concentrations did not differ significantly between asthma patients and controls or between the exacerbation and remission groups (Table 3 and Table 4). Similar to our findings, Mitchell et al. reported no significant difference in plasma IL-33 levels between healthy controls and patients with allergic asthma. However, they found that sputum IL-33 levels were significantly higher in patients with allergic asthma than in healthy controls [24].

This apparent discrepancy can be explained by IL-33’s unique biology as a nuclear alarmin cytokine that acts primarily within local tissue microenvironments [5,8]. IL-33 is rapidly released upon cellular injury and binds with high affinity to ST2 receptors, resulting in its rapid clearance from the systemic circulation [8,10]. There is increasing evidence suggesting that measurements of IL-33 in airway tissue, sputum or bronchoalveolar lavage more accurately reflect local inflammatory activity than serum levels [9,22,27]. Furthermore, heterogeneity among asthma phenotypes, differences in sampling timing relative to exacerbation onset and methodological variability in ELISA assays may contribute to the inconsistent serum findings reported in clinical studies [28,29]. Therefore, circulating IL-33 alone may have limited utility as a systemic biomarker of asthma activity.

Although the effects of vitamin D supplementation on respiratory infections and chronic diseases are inconsistent according to current evidence from randomized controlled trials (RCTs) and meta-analyses of RCTs [30], numerous meta-analyses have demonstrated a significant association between vitamin D deficiency and the presence and severity of asthma [20,21,31].

Vitamin D deficiency has emerged as an important environmental factor that influences asthma susceptibility and exacerbation risk [15,17]. In addition to its traditional endocrine functions, vitamin D has profound immunomodulatory effects, including the suppression of dendritic cell maturation, the inhibition of B-cell proliferation, the promotion of regulatory T-cell differentiation and the enhancement of anti-inflammatory cytokine production [15,16]. In line with previous reports [21,32,33] our study found that vitamin D levels were significantly lower in asthma patients than in healthy individuals and decreased further during exacerbation periods (Figure 2). Vitamin D deficiency has been linked to impaired antiviral immunity and an increased susceptibility to respiratory infections, both of which are major drivers of asthma exacerbations [20].

Importantly, accumulating experimental evidence suggests a direct interaction between vitamin D signaling and the IL-33/ST2 axis. Vitamin D has been shown to increase the production of sST2 by airway epithelial cells and lymphocytes, thereby reducing IL-33-mediated inflammatory signaling [21]. This regulatory mechanism suggests that vitamin D insufficiency may impair endogenous control of alarmin-induced inflammation. The coexistence of reduced vitamin D concentrations and elevated sST2 levels during exacerbations in our cohort may therefore reflect an inadequate compensatory response attempting to restore immune homeostasis under conditions of intensified inflammatory stress.

Our correlation analysis in the patients showed a significant negative correlation was identified between sST2 and 25(OH)D levels (r = −0.333, *p* = 0.016), indicating that lower vitamin D concentrations were associated with higher circulating sST2 levels (Table 5). Additionally, sST2 demonstrated a strong negative correlation with FEV1/FVC ratio, further underscoring its association with disease severity and airway obstruction, as well as a significant positive correlation with WBC count, consistent with systemic inflammatory activation. In contrast, no significant correlation was observed between sST2 and IL-33, or between IL-33 and 25(OH)D.

The significant inverse relationship between sST2 and vitamin D observed in our cohort is consistent with the experimental findings of Pfeffer et al. [21], who demonstrated that vitamin D increases soluble ST2 production in a concentration-dependent manner. Under conditions of vitamin D insufficiency, as observed in our patients whose mean 25(OH)D concentrations were substantially below the 40 ng/mL threshold proposed as necessary for this immunomodulatory effect, the capacity of vitamin D to upregulate sST2 may be impaired. The inverse correlation observed in our data may therefore reflect a compensatory upregulation of sST2 in the context of reduced vitamin D-mediated regulation, rather than a direct stimulatory effect. The absence of a significant correlation between IL-33 and 25(OH)D is consistent with the report by Janeva-Jovanovska et al. [34], who similarly found no significant correlation between vitamin D and IL-33 levels in patients with severe asthma, and may further reflect the limitations of circulating IL-33 as a sensitive systemic biomarker of pathway activation.

For differentiating asthma patients from healthy controls, sST2 demonstrated acceptable discriminatory performance, with a high sensitivity of 92.3% at the optimal cut-off of >2446.1 pg/mL, suggesting its potential utility as a screening biomarker (Figure 3). Notably, for discriminating exacerbation from remission, sST2 showed outstanding performance with an AUC of 0.994, a sensitivity of 100%, and a specificity of 96.0% at a cut-off of >3733 pg/mL. These findings suggest that circulating sST2 may be particularly useful for distinguishing exacerbation from remission rather than for asthma diagnosis alone. This is consistent with the prospective findings of Watanabe et al. [13], who demonstrated that elevated serum sST2 levels predicted severe asthma exacerbation within three months, and further reinforces the potential role of sST2 in precision asthma management.

Consistent with previous literature [35], elevated leukocyte counts and increased IgE levels in asthma patients in our study support ongoing systemic immune activation and allergic sensitization (Table 2).

Eosinophils are major effector cells of type 2 inflammation and play a central role in the IL-33/ST2 signaling pathway in asthma. IL-33 released from damaged airway epithelial cells activates ILC2s, Th2 lymphocytes, mast cells, and eosinophils via the ST2 receptor, promoting IL-5, IL-9, and IL-13 production and eosinophilic airway inflammation [8,11]. Experimental studies have shown that inhibition of IL-33/ST2 signaling reduces eosinophilic inflammation and airway hyperresponsiveness [9].

In our study, consistent with Tanaka et al. [35], eosinophil counts did not differ significantly among the groups (Table 3). Although eosinophilia is a hallmark of type 2 asthma, inhaled corticosteroid therapy has been shown to reduce peripheral eosinophil counts in asthma patients [36]. Since our patients were receiving inhaled corticosteroids, the lack of significant intergroup differences may be related to the suppressive effects of treatment. Therefore, circulating eosinophil counts alone may not fully reflect local airway inflammation or activation of the IL-33/sST2 axis.

From a translational perspective, our findings highlight the potential clinical relevance of the IL-33/ST2 axis as a biomarker pathway and therapeutic target. In the biologics era, asthma management is increasingly targeting upstream epithelial mediators, and therapies directed against IL-33 signaling have demonstrated promising reductions in exacerbation frequency across diverse inflammatory phenotypes [25,26,37]. Identifying accessible biomarkers that reflect pathway activation could therefore facilitate patient stratification and support precision medicine approaches.

### 4.1. Study Limitations

Several limitations of the present study should be acknowledged. First, the single-center design and relatively modest sample size may limit the external generalizability of the findings, and replication in larger multicenter cohorts is required. Second, local airway measurements of IL-33, such as those obtained from induced sputum or bronchoalveolar lavage, were not performed; such measurements may have provided additional insight into tissue-level inflammatory activity that systemic sampling cannot fully capture. Third, phenotypic subgroup analyses stratifying patients by allergic status, eosinophilic versus non-eosinophilic endotype, or asthma severity step were not performed, which may have limited the interpretability of IL-33/ST2 axis dynamics across distinct disease endotypes. Fourth, the cross-sectional nature of biomarker assessment did not permit longitudinal tracking of sST2, IL-33, or vitamin D concentrations over time, precluding conclusions regarding causal directionality or temporal dynamics. Since the study was conducted during the period of the pandemic, factors related to the pandemic, such as reduced environmental exposure, wearing masks and social distancing, may have influenced inflammatory markers and exacerbation patterns, which could represent a potential source of bias. Additionally, pulmonary function testing was not performed in the healthy control group, precluding a three-group comparison of spirometric parameters and limiting the characterization of the control population in terms of baseline airway function. Finally, the high intra-group variability observed in serum IL-33 measurements, together with the known technical limitations of commercial ELISA assays for IL-33 detection, may have reduced the sensitivity to detect significant between-group differences.

### 4.2. Recommendations for Future Research

Future multicenter prospective studies with larger sample sizes and phenotype-specific stratification are needed to confirm these findings. Longitudinal designs with repeated biomarker measurements would better characterize the temporal dynamics of the IL-33/sST2 axis and its interaction with vitamin D status. Local airway sampling methods and year-round recruitment strategies should be incorporated to minimize tissue-level measurement limitations and seasonal confounding. Vitamin D supplementation trials concurrently assessing sST2 and IL-33 levels would provide valuable mechanistic evidence. Finally, the utility of sST2 as a real-time biomarker in the context of emerging biologic therapies targeting the IL-33/ST2 axis, such as itepekimab and astegolimab, warrants further investigation.

## 5. Conclusions

In conclusion, this study demonstrates that increased circulating sST2 levels and reduced vitamin D concentrations are strongly associated with asthma and exacerbation of the disease, whereas serum IL-33 levels may not accurately reflect systemic inflammatory activity. These results reinforce the growing theory that epithelial alarmin signaling is a key aspect of asthma pathophysiology, suggesting that the IL-33/ST2 axis could be used as a diagnostic tool and a potential treatment option in the field of precision asthma medicine. Targeting epithelial alarmin signaling pathways together with optimization of vitamin D status may represent promising strategies for improving asthma control. Further large-scale longitudinal studies are warranted to clarify causal relationships and therapeutic implications.

## Figures and Tables

**Table 1:** Demographic characteristics of the study groups.

Variable	Exacerbation (*n* = 27)	Remission (*n* = 25)	Control (*n* = 28)	*p*-Value
Age (years)	39.04 ± 12.87	41.08 ± 10.41	39.93 ± 10.22	>0.05
BMI (kg/m^2^)	26.01 ± 5.08	26.79 ± 4.30	26.15 ± 4.55	>0.05
Female *n* (%)	13 (48.1%)	13 (52.0%)	13 (46.4%)	>0.05
Male *n* (%)	14 (51.9%)	12 (48.0%)	15 (53.6%)	>0.05

**Table 2:** Evaluation of the study parameters for the patient and control groups.

Parameter	Asthma (*n* = 52)	Control (*n* = 28)	*p*
	Mean ± SD	Mean ± SD	
sST2 (pg/mL)	4113.02 ± 1665.43	2777.53 ± 959.83	<0.001 ^1,^*
IL-33 (pg/mL)	5.52 ± 9.35	2.94 ± 1.05	0.324 ^1^
25(OH)D (ng/mL)	17.07 ± 7.88	31.01 ± 15.64	<0.001 ^1,^*
IgE (IU/mL)	252.71 ± 301.87	96.91 ± 183.8	0.001 ^1,^*
WBC (10^3^/uL)	8.45 ± 1.96	7.25 ± 1.95	0.010 ^2,^*
Eosinophil %	3.14 ± 2.72	2.23 ± 1.31	0.226 ^1^
Eosinophil count (10^3^/uL)	0.30 ± 0.27	0.17 ± 0.11	0.053 ^1^

^1^ Mann–Whitney U test; ^2^ Student’s *t*-test * *p* < 0.05. sST2, the soluble isoform of ST2; IL-33, interleukin-33; 25(OH)D, 25-hydroxyvitamin D; IgE, immunoglobulin E.

**Table 3:** Study parameters of the exacerbation, remission and control groups.

Parameter	Exacerbation (*n* = 27)	Remission (*n* = 25)	Control (*n* = 28)	*p*
	Mean ± SD (Median)	Mean ± SD (Median)	Mean ± SD (Median)	
sST2 (pg/mL)	5256.92 ± 1550.89 (4827.2)	2877.61 ± 475.82 (2773.9)	2777.53 ± 959.83 (2593.8)	<0.001 ^1,^*
IL-33 (pg/mL)	6.24 ± 9.88 (3.1)	4.73 ± 8.87 (2.8)	2.94 ± 1.05 (3)	0.502 ^1^
25(OH)D (ng/mL)	14.68 ± 6.16 (13.3)	19.64 ± 8.81 (16.4)	31.01 ± 15.64 (26.7)	<0.001 ^1,^*
IgE (IU/mL)	329.12 ± 359.74 (175)	170.18 ± 199.44 (57)	96.91 ± 183.8 (34.9)	0.001 ^1,^*
WBC (10^3^/uL)	9.1 ± 2.08 (8.8)	7.76 ± 1.59 (7.4)	7.25 ± 1.95 (7.2)	0.002 ^2,^*
Eosinophil %	3.05 ± 2.94 (2.5)	3.23 ± 2.51 (2.2)	2.23 ± 1.31 (1.8)	0.399 ^1^
Eosinophil # (10^3^/uL)	0.3 ± 0.28 (0.2)	0.29 ± 0.27 (0.2)	0.17 ± 0.11 (0.1)	0.146 ^1^

^1^ Kruskal–Wallis Test; ^2^ one-way ANOVA Test * *p* < 0.05. sST2, the soluble isoform of ST2; IL-33, interleukin-33; 25(OH)D, 25-hydroxyvitamin D; IgE, immunoglobulin E; #, number.

**Table 4:** Evaluation of the exacerbation and remission groups in terms of study parameters.

Parameter	Exacerbation (*n* = 27)	Remission (*n* = 25)	*p*-Value
sST2 (pg/mL)	5256.92 ± 1550.89	2877.61 ± 475.82	<0.001
IL-33 (pg/mL)	6.24 ± 9.88	4.73 ± 8.87	NS
Vitamin D (ng/mL)	14.68 ± 6.16	19.64 ± 8.81	0.038
WBC (10^3^/uL)	9.1 ± 2.08	7.76 ± 1.59	0.012
FEV1/FVC (%)	77.14 ± 18.62	93.47 ± 5.51	<0.001

sST2, the soluble isoform of ST2; IL-33, interleukin-33; 25(OH)D, 25-hydroxyvitamin D; NS, non-significant.

**Table 5:** Pearson correlation coefficients between biomarkers and clinical parameters of patients.

Parameter	IL-33 (pg/mL)	25(OH)D (ng/mL)	sST2 (pg/mL)
	r (95% CI) *p*-value	r (95% CI) *p*-value	r (95% CI) *p*-value
IL-33 (pg/mL)	-	0.190 (−0.088 to 0.440)/0.178	0.272 (−0.001 to 0.508) 0.051
25(OH)D (ng/mL)	0.190 (−0.088 to 0.440)/0.178	-	−0.333 (−0.556 to −0.066) 0.016 *
IgE (IU/mL)	0.120 (−0.158 to 0.381) 0.396	0.077 (−0.200 to 0.343) 0.588	−0.008 (−0.280 to 0.266) 0.955
WBC (10^3^/µL)	−0.017 (−0.289 to 0.257) 0.902	−0.266 (−0.503 to 0.007) 0.056	0.285 (0.013 to 0.518) 0.041 *
Eosinophil %	0.047 (−0.229 to 0.316) 0.741	0.024 (−0.251 to 0.295) 0.868	−0.150 (−0.407 to 0.128) 0.287
FEV1/FVC (%)	0.117 (−0.216 to 0.425) 0.492	0.229 (−0.103 to 0.515) 0.173	−0.640 (−0.799 to −0.399) <0.001 *

* *p* < 0.05. 95% CI, 95% confidence interval; sST2, soluble ST2; 25(OH)D, 25-hydroxyvitamin D; WBC, white blood cell count.

**Table 6:** Logistic regression analysis of parameters associated with asthma and disease exacerbation.

**Parameters Associated with Asthma**				
	95% Confidence Interval			
**Step 1**	**Odds Ratio**	**Lower Bound**	**Upper Bound**	***p***
sST2 (pg/mL)	1.001	1.000	1.001	0.025 *
25(OH)D (ng/mL)	0.870	0.795	0.953	0.003 *
IgE (IU/mL)	1.003	1.000	1.006	0.049 *
**Parameters Associated with Asthma Exacerbation**				
**Step 2**	**Odds Ratio**	**Lower Bound**	**Upper Bound**	***p***
sST2 (pg/mL)	1.012	0.997	1.029	0.046 *

Variables entered into the Step 1 model: sST2, 25(OH)D, IgE, and WBC. Nagelkerke R^2^ = 0.546; model accuracy = 82.5%. Variables entered into the Step 2 model: sST2, 25(OH)D, WBC, and FEV_1_/FVC. Nagelkerke R^2^ = 0.929; model accuracy = 96.2%. * *p* < 0.05. OR, odds ratio; 95% CI, 95% confidence interval; sST2, soluble ST2; 25(OH)D, 25-hydroxyvitamin D; WBC, white blood cell count.

## Data Availability

The datasets generated during the current study are available from the corresponding author upon request.
